# Negative emotionality and behavioral inhibition are associated with subjective frustration after a controlled frustration-induction task

**DOI:** 10.1038/s41598-026-70146-9

**Published:** 2026-09-08

**Authors:** Hannaneh Yazdi, Maria de la Paz Celorio-Mancera, Johan N. Lundström

**Affiliations:** 1 Department of Clinical Neuroscience, Karolinska Institutet, Stockholm, Sweden; 2 Department of Physiology & Pharmacology, Karolinska Institutet, Stockholm, Sweden; 3 Department of Ecology, Environment and Plant Sciences, Stockholm University, Stockholm, Sweden

**Keywords:** Frustration, Emotion regulation, Personality traits, Behavioral tendencies, Individual differences, Self-regulation, Psychology, Human behaviour

## Abstract

Frustration responses vary substantially across individuals, yet the psychological characteristics associated with task-evoked frustration remain unclear. We examined whether four theoretically organized psychological domains, Big Five, BIS/BAS, Self-regulation, and Self-evaluation, were associated with post-task subjective frustration following a controlled frustration-induction task. The final sample comprised 145 participants. Hierarchical regressions with HC3-robust inference first accounted for baseline mood, energy, hunger, and sleep, followed by task-familiarity controls. Baseline-state and familiarity blocks were not significant in the primary models. When entered separately after these controls, each psychological-domain block showed a significant incremental association with frustration at the unadjusted 0.05 level. At the individual-measure level, higher negative emotionality and behavioral inhibition were positively associated with frustration and remained significant in Cook’s-distance sensitivity analyses. No other individual psychological coefficients were significant in the primary robust models. These exploratory findings provide a focused basis for future confirmatory research on individual differences in subjective responses to goal obstruction.

**Supplementary Information:**

The online version contains supplementary material available at https://doi.org/10.1038/s41598-026-70146-9.

## Introduction

Emotions can shape goal-directed behavior by influencing intentions, planning, effort, and responses to goal outcomes^1^. Positive affect may support continued engagement, whereas obstacles and setbacks can evoke negative affective responses such as frustration. Frustration is a negative affective response that commonly arises when goal pursuit is obstructed or when expected outcomes or rewards are not obtained^2–4^. Individuals differ substantially in how they experience and respond to frustration. Similar obstacles may be accompanied by marked irritation or disengagement in some individuals but continued persistence in others. Such variation motivates examination of personality, motivational sensitivity, and self-regulatory capacity as potential correlates of frustration. The present study examined whether selected personality, motivational, self-regulatory, and self-evaluative measures were associated with post-task subjective frustration following the Frustration Induction Task, a controlled task designed to elicit frustration^5^.

The classic frustration-aggression hypothesis linked interference with expected goal attainment to aggression, whereas later reformulations proposed that frustration increases aggressive inclinations insofar as goal obstruction evokes negative affect^3,4^. More broadly, frustration arises within goal pursuit when motivation to obtain an outcome is met by obstruction or nonreward^6^. Responses to obstruction may involve continued approach, increased effort, withdrawal, or disengagement, making frustration relevant to the self-regulation of affect and action^6,7^. Understanding frustration as a goal-related affective process provides valuable insight into how individuals adapt to challenges and setbacks, bridging individual differences in emotional appraisal and emotion regulation. Experimental evidence indicates that blocking access to a valued reward increases subjective frustration and behavioral response vigor, particularly when greater effort has already been invested, or the reward is perceived as close^3,8,9^. Emotion regulation can occur at multiple points in the emotion-generative process, from situation selection and attentional deployment to cognitive change and response modulation^10–12^. Within this framework, frustration offers a relevant context for examining individual differences because goal obstruction can engage negative affect, approach-avoidance motivation, and behavioral control^6,7^. The study did not test specific emotion-regulation strategies. Instead, it examined whether four theoretically organized psychological domains: Big Five, BIS/BAS, Self-regulation, and Self-evaluation, were associated with post-task subjective frustration following a controlled frustration-induction task.

Personality traits have been associated with variation in everyday emotional experience, although such associations may depend on the context in which emotions are elicited^13^. Individuals also differ in their habitual use of emotion-regulation strategies, and these differences are associated with patterns of positive and negative affect^14^. On this basis, the present study considered four complementary domains that may be associated with responses to blocked goals, including Big Five, BIS/BAS, self-regulatory, and self-evaluation.

Big Five traits provide a broad dispositional framework for examining variation in affective and regulatory responding^13,15^. Negative emotionality was of particular interest because the closely related neuroticism domain is strongly associated with negative affectivity and distress-related symptoms^16^, and meta-analytic evidence links higher neuroticism to less adaptive emotion-regulation strategies^15^. Conscientiousness was also theoretically relevant because it has been associated with greater recovery from negative emotional stimuli, although this evidence concerns emotional recovery rather than frustration specifically^17^. The same meta-analysis associated higher extraversion, agreeableness, and openness with greater use of typically adaptive and lower use of typically maladaptive emotion-regulation strategies, although these associations were not specific to frustration^15^. These findings provide a stronger frustration-related rationale for negative emotionality and conscientiousness than for the remaining traits; nevertheless, the complete Big Five domain was examined as a coherent framework for broad personality variation.

Beyond the Big Five, three additional domains were considered: BIS/BAS, Self-regulation, and Self-evaluation. The BIS/BAS domain concerned motivational sensitivity within the Behavioral Inhibition System and Behavioral Activation System framework. The Carver and White BIS/BAS Scales were developed to assess dispositional sensitivities to anticipated punishment and reward: higher BIS sensitivity was associated with greater nervousness during anticipated punishment, whereas BAS Reward Responsiveness and Drive were associated with greater happiness during anticipated reward^18^. Because frustration involves interruption of ongoing goal pursuit, approach–avoidance systems provide a theoretically relevant framework for examining variation in affect and action during obstruction^7^. Stronger BAS drive was tentatively expected to be associated with lower frustration because approach orientation may support continued goal engagement, although this expectation was less direct than the rationale for BIS^7,18^. Accordingly, BIS and the three BAS dimensions were considered together as a motivational-sensitivity domain, while the Carver and White scales were not interpreted as a comprehensive measure of revised Reinforcement Sensitivity Theory.

The Self-regulation domain concerned individual differences in maintaining goal-directed behavior and regulating affect and action under emotional demand. Two dimensions of emotion-regulation difficulty, problems maintaining goal-directed behavior and controlling impulses when distressed, directly concern behavioral regulation under negative affect^19^. Emotional intelligence was relevant because meta-analytic evidence has associated higher emotional intelligence with greater use of generally adaptive and lower use of generally maladaptive emotion-regulation strategies^20^. Internal control was included as a perceived-agency construct, reflecting the belief that outcomes are contingent on one’s own behavior^21^. Although internal control is conceptually distinct from emotion-regulation ability, it was included because perceived agency may be relevant when progress toward a goal is obstructed. Together, these measures were treated as the Self-regulation domain because they capture complementary aspects of behavioral regulation, emotional competence, and perceived agency during demanding goal pursuit.

The Self-evaluation domain included self-evaluative and discomfort-related dispositions. Frost et al. identified concern over mistakes and personal standards as distinct dimensions of perfectionism^22^. Self-esteem was included as a global self-evaluative construct^23^ and has previously been examined as a moderator of appraisal and reactivity during laboratory stress^24^. Discomfort intolerance reflects intolerance of difficulties and hassles^8^. These measures were considered together because they capture complementary aspects of self-evaluation, reactions to mistakes, personal standards, and the perceived tolerability of effort and difficulty.

We predicted that higher post-task subjective frustration would be associated with traits such as negative emotionality, BIS, discomfort intolerance, concern over mistakes, and greater difficulty with impulse control and goal-directed behavior. Conversely, lower post-task frustration was expected to be linked to traits such as conscientiousness, emotional intelligence, and BAS drive.

 and BAS drive. The primary aim was to examine whether the four theoretically organized psychological domains were associated with post-task subjective frustration following controlled goal obstruction. Identifying psychological characteristics associated with post-task subjective frustration may inform future research on individual differences in responses to controlled goal obstruction. The study was observational with respect to individual differences and therefore does not establish that these characteristics causally influence frustration. The findings therefore provide an exploratory basis for future confirmatory research.

## Methods

### Participants

The sample initially comprised 147 participants. Two were excluded, one because of technical difficulties and one because the participant withdrew, yielding a final sample of 145 participants. The final sample comprised 100 females (69.0%) and 45 males (31.0%), aged 18 to 66 years (*M*_*age*_ = 28.38, SD_age_ = 9.11). A priori power considerations were based on the planned association and regression analyses. For bivariate associations, assuming α = 0.05, 80% power, and an expected small-to-medium association of *r* = .25, approximately 123 participants would be required. For the hierarchical regression models, assuming α = 0.05, 80% power, and a medium effect size of Cohen’s f² = 0.15, the required sample size ranged from approximately 91 to 115 participants depending on the variables included in each model. The available sample sizes, *N* = 145 for descriptive analyses, therefore exceeded these target sample sizes. The study was not powered to detect small effects, and exploratory analyses were interpreted accordingly. A descriptive summary of the sample characteristics is provided in Table 1.

**Table 1:** Participant characteristics and descriptive statistics for study variables.

Measure		M	SD	Median (Q1-Q3)
**Participant characteristics**				
Female (n = 100, 69.0%)				
Male (n = 45, 31.0%)				
Age, years		28.38	9.11	27.00 (21.00-32.00)
Post-task subjective frustration		5.40	1.42	5.68 (4.90-6.40)
**Baseline state**				
Mood		5.33	1.02	5.35 (4.78-5.98)
Energy		4.66	1.23	4.90 (3.52-5.44)
Hunger		2.84	1.61	2.50 (1.42-4.36)
Sleep quality		4.56	1.55	4.78 (3.25-5.74)
**Task familiarity**				
Stockholm familiarity		3.67	1.58	3.52 (2.38-5.02)
Device/tablet familiarity		5.20	1.41	5.32 (4.54-6.28)
Navigation-app familiarity		5.88	0.91	6.04 (5.26-6.52)
Gaming frequency		3.02	1.84	2.44 (1.24-4.72)
ABC-keyboard familiarity		2.81	2.18	1.60 (1.06-4.84)
**Big five traits**				
Extraversion		3.07	0.75	3.08 (2.52-3.51)
Agreeableness		3.70	0.67	3.72 (3.31-4.20)
Conscientiousness		3.45	0.74	3.41 (2.99-4.05)
Negative emotionality		2.90	0.94	2.97 (2.20-3.61)
Open-mindedness		3.86	0.61	3.91 (3.41-4.37)
**BIS/BAS**				
BIS		3.00	0.50	3.12 (2.68-3.35)
BAS reward responsiveness		3.36	0.47	3.44 (3.12-3.75)
BAS drive		2.80	0.57	2.84 (2.36-3.20)
BAS fun seeking		2.92	0.61	3.02 (2.52-3.38)
**Self-regulation**				
Internal control		2.85	0.78	2.81 (2.29-3.37)
Difficulties engaging in goal-directed behavior		3.32	0.69	3.38 (2.92-3.82)
Impulse-control difficulties		2.39	0.59	2.31 (1.85-2.83)
Emotional intelligence		4.35	0.97	4.42 (3.82-4.96)
**Self-evaluation**				
Concern over mistakes		2.39	0.87	2.36 (1.78-3.06)
Personal standards		3.32	0.71	3.39 (2.93-3.78)
Discomfort intolerance		2.29	0.63	2.25 (1.78-2.71)
Self-esteem		2.25	0.26	2.25 (2.10-2.40)

*Note. *Female and male entries are reported as *n* (%).; Continuous variables are reported as means (M), standard deviations (SD), and medians with first and third quartiles (Q1–Q3). BIS = Behavioral Inhibition System; BAS = Behavioral Activation System.

### Recruitment

Participants were recruited through online platforms and community advertisements via the Karolinska Institutet Psychology Testing Recruitment System between 21 November and 19 December 2024. Participants received compensation of 50 or 100 SEK in the form of a gift card, contingent on task performance, and the performance-based reward structure was explained before consent. To minimize expectancy effects, the specific purpose of inducing frustration was not disclosed before the task. Recruitment and consent materials instead described the study as a behavioral investigation of the usability of a navigation application, and participants were informed that they would use a navigation program and report on its usability and their experience. After completing the study, participants were fully debriefed about the true purpose of the experiment and were given the opportunity to withdraw their data. The study was approved by the Swedish Ethical Review Authority (Dnr 2024-04648-01). All participants provided written informed consent, and all procedures were conducted in accordance with the Declaration of Helsinki.

### Procedures

**Fig. 1:**
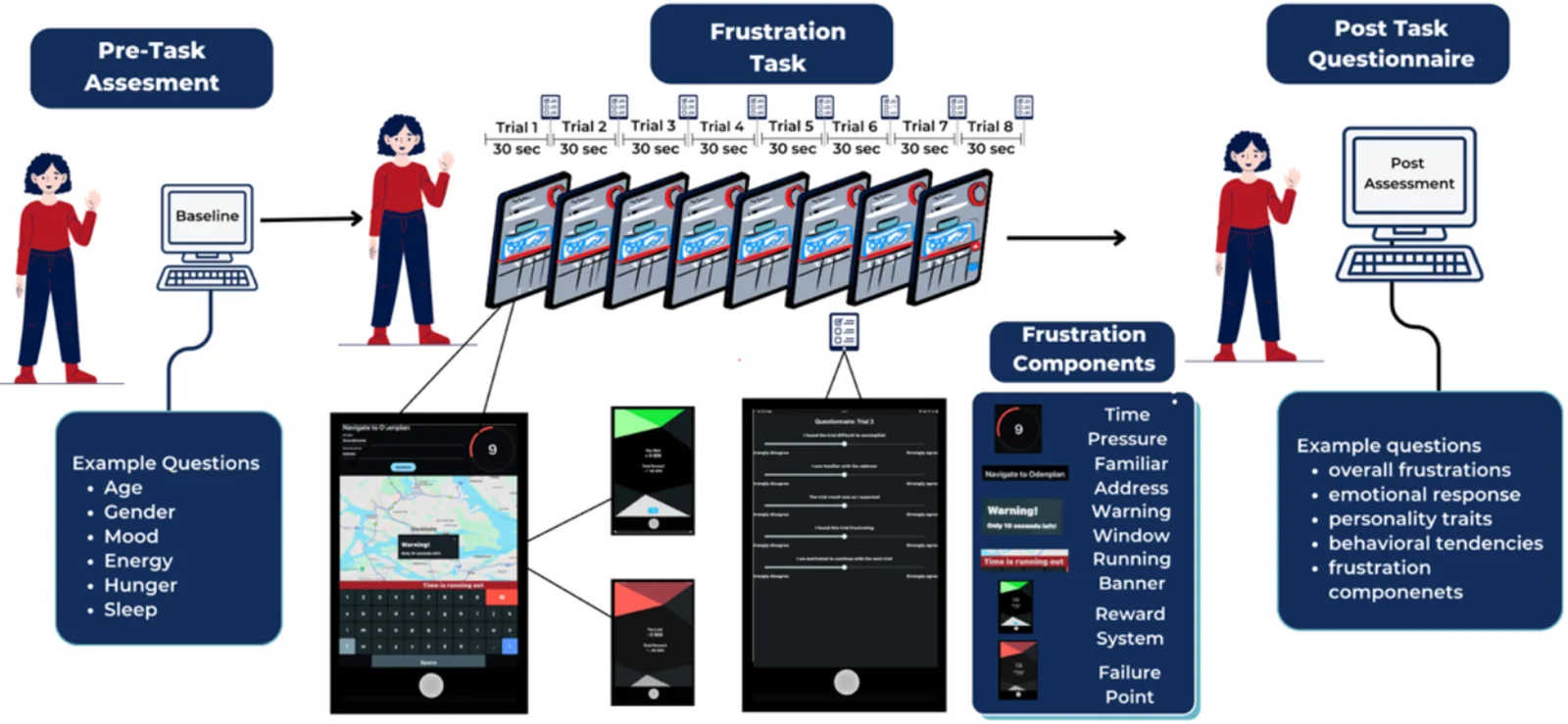
Schematic overview of the study procedures.

Testing took place in a controlled laboratory setting and comprised three phases. During the pre-task assessment, participants reported demographic information, baseline mood, energy, hunger, sleep quality, and task-relevant familiarity. Participants then completed the tablet-based frustration-induction task. Immediately afterward, they rated their overall task-related frustration and emotional responses and completed the psychological questionnaires. Figure 1 summarizes the study procedure.

### Frustration-induction paradigm

Frustration was experimentally induced using the *Frustration Induction Task* (FIT)^5^, a tablet-based paradigm to elicit frustration in a controlled laboratory environment by integrating multiple frustration component triggers. Participants completed a navigation-style address-entry task using an alphabetical (ABC) keyboard layout rather than the standard QWERTY configuration, which disrupted motor fluency and increased cognitive load. The task incorporated 30-second time limits, keyboard disruptions, ambiguous or delayed feedback, warning and running banners, a performance-contingent reward system, and negative performance feedback. These manipulations were designed to obstruct goal progress and reduce perceived control during task performance.

### Measures

To reduce demand characteristics and expectancy effects, personality trait and behavioral tendency questionnaires were administered after the frustration-induction task. Conducting these measures prior to the task could have increased participants’ awareness of the study’s focus on frustration and emotion regulation, thereby introducing expectancy effects that may have influenced task engagement and emotional responses. This ordering was therefore selected to preserve the integrity of the frustration manipulation, while acknowledging that it limits temporal inference. Although these instruments were selected to assess relatively stable individual differences, their post-task administration means that task-related state influences cannot be excluded.

#### Baseline state and task familiarity

Before the task, participants rated their current mood, physical energy, hunger, and previous-night sleep quality using single-item 7-point scales. Participants also rated familiarity with Stockholm streets or neighborhoods, iPads or other tablets, navigation applications, and the ABC keyboard on 7-point scales. Gaming was assessed as frequency of computer-game play from 1 (never) to 7 (always). Higher scores indicated greater levels of the corresponding baseline state or familiarity. These nine variables were included as control variables in the hierarchical regression models. The following items were presented: *“Rate your current mental mood”* (1 = Very poor to 7 = Excellent), *“Rate your current level of physical energy”* (1 = Completely drained to 7 = Fully energized), *“Rate your current level of hunger”* (1 = Not hungry at all to 7 = Very hungry), *“Rate your quality of sleep last night”* (1 = Very poor to 7 = Excellent). Participants also reported their familiarity with tools and tasks that may affect frustration sensitivity: *“How familiar are you with the ABC keyboard?”* (1 = Not familiar at all to 7 = Very familiar), *“How familiar are you with navigation apps (e.g.*,* Google Maps*,* Apple Maps*,* Waze)?”* (1 = Not familiar at all to 7 = Very familiar), *“How often do you play computer games?”* (1 = Never to 7 = Always), *“How familiar are you with the use of iPads or other tablets?”* (1 = Not familiar at all to 7 = Very familiar). These baselines and familiarity measures were collected immediately before the experimental task and designed to control for individual differences that could modulate frustration responses.

#### Post-task subjective frustration

Post-task subjective frustration was assessed immediately after the FIT. Before responding, participants were provided with the following definition: “In this study, we define frustration as a negative emotional response that occurs when an individual is unable to achieve a desired goal or is blocked from attaining a particular outcome.” Participants then responded to the item: “According to the definition of frustration above, rate the level of frustration that you experienced from performing the task as a whole.” Responses were recorded on a 7-point Likert scale ranging from 1 (not frustrated) to 7 (very frustrated). The single-item response was used directly as the sole primary outcome, with higher scores indicating greater post-task subjective frustration. It was not combined with any personality or behavioral measure.

#### Big five traits

The 15-item Big Five Inventory-2 Extra-Short Form (BFI-2-XS)^25^ assessed extraversion, agreeableness, conscientiousness, negative emotionality, and open-mindedness, with three items per domain. Items were rated from 1 (disagree strongly) to 5 (agree strongly).

#### Behavioral inhibition and activation

The Carver and White BIS/BAS Scales^18^ assessed behavioral inhibition (BIS; 7 items), BAS reward responsiveness (5 items), BAS drive (4 items), and BAS fun seeking (4 items). Responses ranged from 1 (strong agreement) to 4 (strong disagreement) and were recoded so that higher scores represented greater inhibition or activation. Subscale scores were item means. One BAS drive item had been stored on a 1–7 response scale; it was linearly rescaled to the intended 1–4 metric before scoring. These scales were used as measures of the original BIS/BAS approach–avoidance constructs and not as a comprehensive operationalization of revised Reinforcement Sensitivity Theory.

#### Self-regulation measures

Internal control was assessed using the seven-item Internal Control subscale of Levenson’s locus-of-control measure^21^, rated on a six-point agreement scale. Difficulties engaging in goal-directed behavior (5 items) and impulse-control difficulties (6 items) were assessed using the corresponding subscales of the Difficulties in Emotion Regulation Scale^19^, with responses from 1 (almost never) to 5 (almost always). Emotional intelligence was assessed using five selected items from the Emotional Skills and Competence Questionnaire^26^, rated on a five-point scale. Reverse-keyed items were recoded before composite scoring. Higher scores indicated greater internal control, goal-directed difficulties, impulse-control difficulties, or emotional intelligence, respectively.. 

#### Self-evaluation measures

Perfectionistic concern over mistakes (9 items) and personal standards (7 items) were assessed with subscales of the Frost Multidimensional Perfectionism Scale^22^, using five-point agreement ratings. Discomfort intolerance was assessed with the eight-item Discomfort Intolerance subscale of the Frustration Discomfort Scale^8^, rated from 0 (absent) to 4 (very strong). Self-esteem was assessed with the 10-item Rosenberg Self-Esteem Scale^23^, using a four-point response format. Reverse-keyed items were recoded before composite scoring.Higher scores indicated greater concern over mistakes, personal standards, discomfort intolerance, or self-esteem, respectively. 

#### Emotional response measure

Post-task emotional responses were assessed using the 20 emotion adjectives from the Positive and Negative Affect Schedule (PANAS)^27^, a widely validated self-report instrument for assessing state affect. The PANAS consists of 20 emotion adjectives, divided into two 10-item subscales: Positive Affect (PA), including active, alert, interested, attentive, determined, strong, enthusiastic, proud, inspired, and excited, and Negative Affect (NA), including upset, distressed, jittery, irritable, nervous, ashamed, hostile, guilty, afraid, and scared. Consistent with previous validation studies^27^, internal consistency in the present sample was good (PA: Cronbach’s α = 0.84; NA: Cronbach’s α = 0.89). Participants rated “Rate the existence of each of the following emotions in relation to your experience of frustration during the task” on a 5-point Likert scale ranging from 1 (very slightly or not at all) to 5 (extremely).

### Scale reliability

Cronbach’s alpha and Feldt 95% confidence intervals were calculated from the item-level data for each multi-item psychological measure, as shown in Table 2. Most measures showed acceptable to high internal consistency. Reliability was low for emotional intelligence (*α* = 0.55), extraversion (*α* = 0.53), agreeableness (*α* = 0.46), conscientiousness (*α* = 0.52), and open-mindedness (*α* = 0.35); results involving these measures were therefore interpreted cautiously. Negative emotionality showed acceptable reliability (*α* = 0.78).

**Table 2:** Internal consistency reliability of multi-item measures.

Scale	Items	α	95% CI
Internal control	7	0.70	[0.62, 0.77]
Difficulties engaging	5	0.90	[0.87, 0.92]
Impulse-control difficulties	6	0.89	[0.86, 0.91]
Emotional intelligence	5	0.55	[0.42, 0.66]
Concern over mistakes	9	0.89	[0.86, 0.92]
Personal standards	7	0.82	[0.77, 0.86]
Discomfort intolerance	8	0.83	[0.78, 0.87]
Self-esteem	10	0.87	[0.83, 0.90]
BIS	7	0.83	[0.78, 0.87]
BAS reward responsiveness	5	0.88	[0.85, 0.91]
BAS drive	4	0.83	[0.79, 0.87]
BAS fun seeking	4	0.84	[0.79, 0.88]
Extraversion	3	0.53	[0.37, 0.65]
Agreeableness	3	0.46	[0.28, 0.59]
Conscientiousness	3	0.52	[0.36, 0.64]
Negative emotionality	3	0.78	[0.70, 0.83]
Open-mindedness	3	0.35	[0.15, 0.52]

Note. Confidence intervals are Feldt 95% confidence intervals. The post-task subjective frustration outcome and the baseline and familiarity controls were single items and therefore do not have internal-consistency coefficients. *α* = Cronbach’s alpha; BIS = Behavioral Inhibition System; BAS = Behavioral Activation System.

### Data analysis

Analyses were restricted to the manipulated condition because the research question concerned individual differences following exposure to the frustration induction. Descriptive analyses used all available observations in that condition (*N* = 145). One participant had missing mood and sleep values; complete-case analysis therefore yielded *N* = 144 for every regression model.Because of the REDCap configuration used for data collection, response values were stored as integers multiplied by 100 (e.g., 100–500 represented 1.00–5.00). This storage convention was used to enable fine-grained response values while avoiding issues with decimal rounding in the REDCap interface. Before scoring or analysis, these values were divided by 100 to restore their intended numeric scale. Reverse-keyed Big Five items were recoded, and each Big Five trait score was calculated as the mean of its three items; higher scores indicated more of the named trait. BIS/BAS items were oriented so that higher scores indicated more of the construct and were averaged on the 1–4 metric.

Hierarchical ordinary least-squares regression was the primary analysis, with post-task subjective frustration as the outcome. Mood, energy, hunger, and sleep quality were entered in Block 1 Familiarity with Stockholm, devices/tablets, navigation applications, gaming frequency, and the ABC keyboard were added in Block 2 Four separate parallel Block 3 models then added one psychological domain at a time: (A) Big Five traits, (B) BIS/BAS, (C) self-regulation, or (D) self-evaluation. The four Block 3 models were not successive steps in a single cumulative model.

Because the nonconstant-variance score test indicated heteroskedasticity, inference was based on HC3 heteroskedasticity-robust standard errors, 95% confidence intervals, and Wald *F* tests for each added block. Model fit was summarized using *R*², adjusted *R*², and Δ*R*². Variance-inflation factors were examined for multicollinearity. Cook’s distance greater than 4/*N* was used as a model-specific screening guideline for sensitivity analyses; screened cases were retained in all primary analyses and excluded only from the corresponding sensitivity model. Pearson correlations were calculated as secondary descriptive associations between post-task subjective frustration, psychological measures, and emotion ratings. All tests were two-sided with *α* = 0.05. Because the domain and correlation analyses were exploratory, no multiplicity adjustment was applied; interpretation emphasized effect estimates, confidence intervals, and sensitivity results. Analyses were conducted in R version 4.4.1 within RStudio version 2026.8.2.200,using the readr, dplyr, psych, sandwich, lmtest, and car packages.

## Results

### Hierarchical regression analyses

Block-level results are presented in Table 3, and the full psychological coefficients estimates, including nonsignificant associations are presented in Table 4. Separate hierarchical multiple regression models were conducted to examine whether theoretically grouped psychological characteristics were associated with post-task subjective frustration after accounting for baseline state and task-familiarity variables. Baseline mood, energy, hunger, and sleep were entered in Block (1) Task-familiarity variables were entered in Block (2) Each psychological domain was then entered separately in Block (3) Tests of regression assumptions indicated heteroskedasticity in all models. Statistical inference was therefore based on HC3 robust standard errors, confidence intervals, and robust Wald tests. Maximum variance-inflation factors were below 2.00 in all models, indicating no material multicollinearity concern. Potentially influential observations were evaluated in sensitivity analyses using Cook’s distance greater than 4/*N* as a screening guideline.

### Regression diagnostics

The nonconstant-variance score test was significant for every model (*p* = .025 for the baseline model and *p* < .001 for the remaining models), supporting the use of HC3 robust inference. Maximum variance-inflation factors ranged from 1.53 to 1.92, indicating no material multicollinearity concern. Cook’s-distance screening identified 8–12 potentially influential cases across models; these observations were retained in the primary analyses. Complete diagnostics are reported in Supplementary Table S1.

### Baseline state

The baseline state block explained 4.8% of the variance in post-task subjective frustration (*R*² = 0.048, adjusted *R*² = 0.021). The HC3 robust block test did not reach statistical significance, *F*(4, 139) = 2.35, *p* = .057. Hunger showed a positive association in the baseline-only model (*B* = 0.150, robust *SE* = 0.070, *p* = .034), but this association weakened after familiarity variables were added and was not interpreted as stable.

### Task familiarity

Adding the five familiarity variables increased explained variance by Δ*R*² = 0.068, yielding *R*² = 0.116 and adjusted *R*² = 0.057. The robust test of the familiarity block was nonsignificant, *F*(5, 134) = 1.95, *p* = .091. In the primary control model, greater device/tablet familiarity was associated with lower frustration (*B* = − 0.207, robust *SE* = 0.084, 95% CI [− 0.373, − 0.041], *p* = .015), whereas greater navigation-app familiarity was associated with higher frustration (*B* = 0.336, robust *SE* = 0.168, 95% CI [0.004, 0.667], *p* = .048). Neither association remained significant in the sensitivity analysis; the familiarity findings were therefore treated as unstable. Complete HC3-robust coefficients for the common baseline and familiarity control model are reported in Supplementary Table S2.

### Big five traits

Adding the Big Five traits increased explained variance by ΔR² = 0.082, yielding R² = 0.199 and adjusted R² = 0.112. The Big Five block was significant, robust *F*(5, 129) = 2.39, *p* = .041. Negative emotionality was positively associated with post-task subjective frustration (*B* = 0.378, robust SE = 0.147, 95% CI [0.087, 0.669], *p* = .011). Extraversion, agreeableness, conscientiousness, and open-mindedness were not significantly associated with frustration (Table 4).

### BIS/BAS

The BIS/BAS block produced the largest increment in explained variance (Δ*R*² = 0.122). The full model explained 23.8% of the variance (*R*² = 0.238, adjusted *R*² = 0.162), and the block was significant, robust *F*(4, 130) = 3.29, *p* = .013. BIS was positively associated with post-task subjective frustration (*B* = 0.789, robust *SE* = 0.315, 95% CI [0.166, 1.412], *p* = .014). BAS reward responsiveness, BAS drive, and BAS fun seeking were not significantly associated with frustration.

### Self-regulation

Adding the self-regulation variables in Block 3 increased the explained variance to *R*² = 0.210, adjusted *R*² = 0.131, corresponding to Δ*R*² = 0.094. The self-regulation block was statistically significant using the robust block test, *F*(4, 130) = 2.60, *p* = .039. However, none of the individual regression coefficients reached statistical significance in the primary robust model. Difficulties engaging in goal-directed behavior showed the strongest association, *B* = 0.475, robust *SE* = 0.247, 95% CI [− 0.014, 0.964], *p* = .057. Internal control, impulse-control difficulties, and emotional intelligence were not significantly associated with frustration. The block remained statistically significant in the sensitivity analysis, but the individual coefficients were not interpreted because they were not significant in the primary model.

### Self-evaluation

Adding the self-evaluation measures increased explained variance by Δ*R*² = 0.066. The full model explained 18.2% of the variance (*R*² = 0.182, adjusted *R*² = 0.100), and the block was significant, robust *F*(4, 130) = 2.92, *p* = .024. No individual measure reached statistical significance in the primary robust model. Concern over mistakes showed the largest coefficient (*B* = 0.306, robust *SE* = 0.161, 95% CI [− 0.013, 0.625], *p* = .059). Personal standards, discomfort intolerance, and self-esteem were not significantly associated with frustration. This finding was also interpreted at the domain level.

**Table 3:** Hierarchical regression block-level results for post-task subjective frustration.

Model/block	*N*	*R*²	Adj. *R*²	ΔR²	Robust Wald F	df	*p*
Block 1: Baseline state	144	0.048	0.021	0.048	2.35	4, 139	0.057
Block 2: Familiarity	144	0.116	0.057	0.068	1.95	5, 134	0.091
Model A: Big Five	144	0.199	0.112	0.082	2.39	5, 129	0.041
Model B: BIS/BAS	144	0.238	0.162	0.122	3.29	4, 130	0.013
Model C: Self-regulation	144	0.210	0.131	0.094	2.60	4, 130	0.039
Model D: Self-evaluation	144	0.182	0.100	0.066	2.92	4, 130	0.024

Note. The baseline and familiarity rows are the common control sequence. Models A–D are separate parallel regressions, each adding one psychological domain to the same baseline and familiarity controls. Wald F and p are HC3 robust Wald tests. For Block 1, ΔR² equals the model R²; for Block 2, ΔR² is relative to Block 1; for Models A–D, ΔR² is relative to the common Block 2 control model.

**Table 4:** HC3 robust coefficients for psychological measures associated with post-task subjective frustration.

Block	Psychological measure	B	Robust SE	95% CI	*p*
Big Five	Extraversion	0.256	0.165	[-0.070, 0.582]	0.122
Big Five	Agreeableness	0.109	0.218	[-0.322, 0.540]	0.617
Big Five	Conscientiousness	− 0.294	0.188	[-0.666, 0.078]	0.121
Big Five	Negative emotionality	0.378	0.147	[0.087, 0.669]	0.011
Big Five	Open-mindedness	− 0.054	0.242	[-0.533, 0.425]	0.824
BIS/BAS	BIS	0.789	0.315	[0.166, 1.412]	0.014
BIS/BAS	BAS reward responsiveness	− 0.254	0.326	[-0.899, 0.391]	0.437
BIS/BAS	BAS drive	0.385	0.223	[-0.056, 0.826]	0.086
BIS/BAS	BAS fun seeking	0.411	0.241	[-0.066, 0.888]	0.090
Self-regulation	Internal control	− 0.262	0.185	[-0.628, 0.104]	0.159
Self-regulation	Difficulties engaging in goal-directed behavior	0.475	0.247	[-0.014, 0.964]	0.057
Self-regulation	Impulse-control difficulties	0.268	0.239	[-0.205, 0.741]	0.264
Self-regulation	Emotional intelligence	− 0.151	0.157	[-0.462, 0.160]	0.339
Self-evaluation	Concern over mistakes	0.306	0.161	[-0.013, 0.625]	0.059
Self-evaluation	Personal standards	− 0.078	0.247	[-0.567, 0.411]	0.752
Self-evaluation	Discomfort intolerance	0.382	0.244	[-0.101, 0.865]	0.121
Self-evaluation	Self-esteem	− 0.208	0.497	[-1.191, 0.775]	0.676

Note. Coefficients are from four separate final hierarchical regression models. Each model was adjusted for mood, energy, hunger, sleep, Stockholm familiarity, device familiarity, navigation-app familiarity, gaming frequency, and ABC-keyboard familiarity, as well as the other psychological measures included in the same domain. Negative emotionality and BIS were the only psychological measures significantly associated with post-task subjective frustration, with *p* < .05 and 95% confidence intervals that excluded zero.

### Sensitivity analyses

Model-specific sensitivity analyses excluded observations with Cook’s distance greater than 4/*N* only from the corresponding sensitivity model; these observations were not classified automatically as outliers. The Big Five, BIS/BAS, self-regulation, and self-evaluation remained significant as presented in Supplementary Table S3. Negative emotionality remained significant with a similar coefficient (*B* = 0.391, *p* = .003), as did BIS (*B* = 0.714, *p* = .002) presented in Supplementary Table S4. The baseline block crossed the 0.05 threshold in the sensitivity model (*p* = .038) and was therefore classified as unstable. Familiarity remained nonsignificant, and the device/tablet and navigation-app coefficients no longer reached significance. Thus, the stable individual psychological associations were limited to negative emotionality and BIS.

### Secondary descriptive associations

As a secondary descriptive analysis, post-task subjective frustration showed positive unadjusted bivariate correlations with BIS (r = .28), difficulties engaging in goal-directed behavior (r = .27), negative emotionality (r = .25), impulse-control difficulties (r = .23), discomfort intolerance (r = .22), BAS fun seeking (r = .22), and concern over mistakes (r = .20), and a negative correlation with conscientiousness (r = −.20). Among the post-task emotion ratings, the largest correlations with frustration were observed for upset (r = .52), irritable (r = .47), distressed (r = .36), jittery (r = .34), and ashamed (r = .33). These correlations were descriptive and were not used to determine the hierarchical regression models.

## Discussion

The experience of frustration is not determined solely by the obstacle itself. Comparable obstacles can produce markedly different subjective experiences across individuals, suggesting that the impact of blocked goal pursuit depends partly on characteristics of the person encountering the obstruction. This view is consistent with theoretical accounts that place frustration within the broader dynamics of goal interruption, nonreward, and behavioral adjustment^6,7,28^. In the present study, the clearest individual differences in post-task subjective frustration were associated with negative emotionality and behavioral inhibition. These characteristics originate from different theoretical traditions, yet their convergence is informative. Negative emotionality concerns a broader disposition toward negative affective experience^16^, whereas behavioral inhibition concerns sensitivity to aversive, uncertain, or conflicting conditions^18^. Their joint prominence therefore raises the possibility that variation in frustration reflects both a tendency toward stronger negative affective experience and greater sensitivity when ongoing goal pursuit becomes aversive or conflicted^30^. Rather than pointing to a broad profile of poor self-regulation, the findings suggest a more specific pattern involving affective disposition and motivational sensitivity that may help explain why the same frustrating situation is experienced differently across individuals.

Negative emotionality may represent one pathway through which an obstructed goal acquires greater subjective emotional weight. Individuals higher in negative emotionality are generally more prone to negative affect, distress, and emotionally aversive experiences^16^, and neuroticism has also been associated with greater reliance on less adaptive forms of emotion regulation^15^. More recent evidence from daily life further suggests that neuroticism is related to how negative affect unfolds around stressful experiences, including differences in emotional reactivity and recovery^29^. Viewed in this context, the present association with frustration may reflect a broader tendency for adverse or obstructive events to acquire greater affective significance in individuals higher in negative emotionality. Importantly, however, frustration is not interchangeable with general negative affect or stress. It arises specifically in relation to blocked goals, unmet expectations, or nonreward. The present finding therefore extends, rather than simply reproduces, the broader literature on neuroticism and negative affect by suggesting that controlled goal obstruction may be one context in which this dispositional sensitivity becomes particularly relevant. Whether this association reflects a stronger initial emotional reaction to obstruction, a more negative appraisal of the event, greater persistence of the resulting affect, or some combination of these processes remains an open question.

Behavioral inhibition offers a different, but potentially complementary, perspective on why goal obstruction may be experienced as frustrating. In the original BIS/BAS framework, BIS reflects dispositional sensitivity to anticipated punishment, with higher BIS associated with stronger negative affect when punishment is expected^18^. Later revisions of Reinforcement Sensitivity Theory shifted the emphasis of BIS toward situations involving motivational conflict rather than aversive stimulation alone, and experimental work has linked individual differences in BIS to responses under approach-avoidance conflict^30^. This distinction is particularly relevant to the present task, which required participants to continue pursuing a performance goal while encountering time pressure, interruptions, ambiguous or negative feedback, and a performance-contingent reward. Such conditions could plausibly create competing pressures to persist toward the goal while also responding to an increasingly aversive situation. Higher BIS may therefore be associated with a more salient or affectively consequential experience of this conflict, which could help explain its relationship with subjective frustration. However, this interpretation remains provisional. The Carver and White BIS scale used here reflects the original BIS/BAS framework and cannot be treated as a direct measure of the conflict system proposed in revised Reinforcement Sensitivity Theory. This distinction is important because contemporary evidence for the neurobehavioral correlates of BIS remains less consistent than that for BAS and appears to depend partly on whether motivational systems are actively engaged by the situation^31^. The absence of clear associations with the three BAS dimensions further suggests that frustration in this context was not simply the inverse of reward or approach sensitivity but may have been more closely related to how individuals respond when ongoing goal pursuit becomes difficult, conflicted, or aversive.

Perhaps equally informative is that several characteristics that appear intuitively relevant to frustration did not emerge as clear individual correlates. Difficulties maintaining goal-directed behavior when distressed, impulse-control difficulties, emotional intelligence, perfectionistic concerns, discomfort intolerance, and self-esteem all describe aspects of how people regulate themselves, evaluate their performance, or tolerate difficult experiences. Yet frustration may not simply reflect a general capacity to regulate emotion or maintain positive self-evaluation. Emotion regulation includes the capacity to continue acting in desired ways despite emotional distress^19^, while emotional intelligence has been linked to flexible regulation across different stages of an emotional experience^20^. These capacities may therefore be more relevant to how frustration is managed, expressed, or sustained than to how intensely it is experienced following a particular episode of goal obstruction. A similar distinction may apply to self-evaluative tendencies. Concern over mistakes captures a particularly strong focus on errors within multidimensional perfectionism^22^, whereas discomfort intolerance reflects difficulty tolerating hassles and uncomfortable demands^8^. Both seem theoretically relevant to a task involving repeated obstruction, performance difficulty, and negative or ambiguous feedback, yet neither emerged as a clear individual correlate of frustration. One possibility is that such characteristics become important only at stages of the frustration response, for example during appraisal, persistence, disengagement, or recovery. Another is that their relevance depends on combinations of characteristics rather than any single disposition. The present findings cannot distinguish between these possibilities, but they argue against a simple account in which stronger frustration is merely a reflection of generally poorer self-regulation or more negative self-evaluation.

These findings can be situated within a broader view of frustration as an affective response to disruption during goal pursuit. Contemporary accounts of frustrative nonreward describe an aversive response that can arise when an expected reward is unexpectedly omitted, reduced, delayed, or made inaccessible, with consequences for motivation, learning, conflict, and subsequent behavior^28,32^. More broadly, self-regulatory accounts link affective experience to ongoing progress toward goals and to approach and avoidance processes, emphasizing that emotion depends partly on how goal pursuit is unfolding rather than on the outcome alone^7^. From this perspective, the present pattern raises the possibility that the same external obstruction can acquire different subjective significance depending on the individual encountering it. Negative emotionality and behavioral inhibition may be relevant to different aspects of this variation: negative emotionality to the tendency to experience adverse events more negatively, and behavioral inhibition to sensitivity when goal pursuit becomes aversive, uncertain, or conflicted. These possibilities are complementary rather than interchangeable, and the present study cannot determine the processes through which either characteristic is related to frustration. It is also important not to equate the present task with a pure frustrative-nonreward paradigm. The task combined several forms of obstruction, including time pressure, disrupted task execution, ambiguous or negative feedback, and performance-contingent reward, whereas frustrative nonreward refers more specifically to violations involving expected appetitive outcomes. The broader implication is therefore not that the present findings demonstrate a frustrative-nonreward mechanism, but that individual differences in affective disposition and behavioral inhibition may help explain why complex goal obstruction carries greater subjective emotional impact for some individuals than for others. An important next question is whether the same pattern appears when frustration arises from different forms of obstruction, such as reward loss, delay, uncontrollable failure, or social interference.

The broader pattern also helps clarify what did not readily account for differences in frustration. Immediate state factors such as mood, energy, hunger, and sleep, as well as familiarity with the devices and task-related activities, did not show stable relationships with post-task frustration. This does not imply that such factors are generally unimportant, but it suggests that the individual variation observed here cannot be readily reduced to participants simply arriving in a poorer state or being less familiar with the task environment. The post-task emotion ratings provide a complementary view of what stronger frustration felt like in this setting. Frustration was most closely accompanied by upset, irritability, distress, jitteriness, and shame, indicating that the experience was embedded in a broader pattern of negative affect rather than occurring as an isolated judgment about task difficulty. At the same time, these emotional associations were descriptive and should not be taken to mean that any particular emotion caused, preceded, or constituted the frustration response itself. Together, these secondary findings help situate the main individual-difference results within the immediate experiential context of the task without offering an alternative explanation for them.

In conclusion, the present findings suggest that individual differences in frustration may be better understood by asking not only what obstructs a goal, but why the same obstruction acquires different emotional significance across individuals. Negative emotionality and behavioral inhibition emerged as the clearest individual characteristics associated with stronger post-task frustration, pointing to potentially distinct but complementary aspects of this variability: a broader disposition toward negative affective experience and heightened sensitivity when goal pursuit becomes aversive or conflicted. This distinction offers a useful direction for future research. Rather than treating frustration as a single end-state, future studies could examine its unfolding over time and test whether negative emotionality is more closely related to the intensity or persistence of the affective response, whereas behavioral inhibition becomes particularly relevant during moments of uncertainty, conflict, or interrupted action. Comparing these patterns across different forms of goal obstruction, such as reward omission, delay, repeated failure, loss of control, or social interference, could further reveal whether the same individual characteristics matter across frustration contexts or whether different obstacles engage different vulnerabilities. A particularly informative next step would be to examine negative emotionality and behavioral inhibition jointly, using repeated subjective, behavioral, and physiological measures to determine whether they characterize different routes to frustration, different phases of the response, or their combination. In this way, the present findings provide a basis for moving from identifying who reports greater frustration toward understanding how individual dispositions shape the emergence, escalation, and resolution of frustration during goal pursuit.

### Limitations and future research

Several limitations should guide interpretation of these findings. First, the psychological questionnaires were administered after the frustration-induction task. Although these instruments were intended to assess relatively stable individual differences, responses may have been influenced by the preceding task experience. The observed relationships should therefore be interpreted as concurrent associations, and temporal precedence between the psychological characteristics and post-task frustration cannot be established. Second, frustration was assessed using a single global post-task rating, and no corresponding pre-task frustration measure was obtained. The outcome therefore provides a direct assessment of subjective frustration following the task, but it does not permit estimation of internal consistency or separation of task-related frustration from any pre-existing individual differences in frustration level. Third, both the outcome and most psychological measures relied on self-report, which may increase shared-method and response biases. Finally, the sample was predominantly female and relatively young, and frustration was elicited within a controlled laboratory task. Generalization to other populations and to frustration arising in different social, occupational, or everyday contexts therefore remains to be established.

## Supplementary Information

Below is the link to the electronic supplementary material.

Supplementary Material 1

## Data Availability

The datasets generated and analyzed during the current study are available from the corresponding author upon reasonable request.
