# Supplementary material for: Negative emotionality and behavioral inhibition are associated with subjective frustration after a controlled frustration-induction task
Source: Sci Rep. 2026 Sep 8;16:28075. doi: 10.1038/s41598-026-70146-9 (PMC13554233; doi:10.1038/s41598-026-70146-9)
Supplement: Supplementary file 1 [file 41598_2026_70146_MOESM1_ESM.docx]

**Supplementary Material**

**Supplementary Table S1**

*Regression Diagnostics for the Primary Models*

| **Model** | **Nonconstant-variance test p** | **Maximum VIF** | **Maximum Cook's D** | **Flagged n** |
| --- | --- | --- | --- | --- |
| Baseline | .025 | 1.53 | .112 | 8 |
| Baseline + familiarity | < .001 | 1.68 | .074 | 11 |
| Big Five | < .001 | 1.92 | .110 | 9 |
| BIS/BAS | < .001 | 1.79 | .131 | 11 |
| Self-regulation | < .001 | 1.90 | .085 | 12 |
| Self-evaluation | < .001 | 1.85 | .081 | 12 |

*Note.* The nonconstant-variance score test was significant for every model, motivating the use of HC3 robust inference. Maximum VIF values were below 2.00, indicating no material multicollinearity concern. The Cook's distance guideline was 4/144 = .0278. Flagged cases were retained in all primary models and excluded only in the corresponding sensitivity analyses.

**Supplementary Table S2**

*HC3 Robust Coefficients for the Baseline and Familiarity Control Model*

| **Control variable** | **B** | **Robust SE** | **95% CI** | **p** |
| --- | --- | --- | --- | --- |
| Mood | .014 | .150 | [-.283, .311] | .927 |
| Energy | -.165 | .128 | [-.418, .089] | .201 |
| Hunger | .133 | .068 | [-.002, .268] | .053 |
| Sleep | .057 | .087 | [-.115, .229] | .511 |
| Stockholm familiarity | .010 | .076 | [-.140, .160] | .895 |
| Device familiarity | -.207 | .084 | [-.373, -.041] | .015 |
| Navigation-app familiarity | .336 | .168 | [.004, .667] | .048 |
| Gaming frequency | .091 | .069 | [-.046, .228] | .190 |
| ABC-keyboard familiarity | .026 | .056 | [-.085, .138] | .639 |

*Note.* N = 144. Coefficients are from the common Block 2 control model. Confidence intervals and p values are based on HC3 robust standard errors. Device familiarity and navigation-app familiarity reached *p* < .05 in the primary model but not in the corresponding Cook's distance sensitivity analysis. The familiarity block was nonsignificant; these individual associations are therefore interpreted as unstable.

**Supplementary Table S3**

*Cook's Distance Sensitivity Analysis of Regression Blocks*

| **Block** | **Flagged n** | **Sensitivity N** | **Sensitivity robust F** | **df** | **Primary p** | **Sensitivity p** | **Interpretation** |
| --- | --- | --- | --- | --- | --- | --- | --- |
| Baseline state | 8 | 136 | 2.62 | 4, 131 | .057 | .038 | Threshold-dependent |
| Familiarity | 11 | 133 | 1.14 | 5, 123 | .091 | .344 | Nonsignificant in both |
| Big Five | 9 | 135 | 3.14 | 5, 120 | .041 | .011 | Significant in both |
| BIS/BAS | 11 | 133 | 3.99 | 4, 119 | .013 | .005 | Significant in both |
| Self-regulation | 12 | 132 | 4.39 | 4, 118 | .039 | .002 | Significant in both |
| Self-evaluation | 12 | 132 | 3.53 | 4, 118 | .024 | .009 | Significant in both |

*Note.* Cases were flagged using Cook's distance > 4/N within each model. Flagged cases were retained in the primary analyses and excluded only from the corresponding model-specific sensitivity analysis. The robust Wald *F* and *df* columns refer to the sensitivity models. The baseline block crossed the .05 threshold; the familiarity block remained nonsignificant; and all four psychological blocks remained below the unadjusted .05 threshold in the sensitivity analyses.

**Supplementary Table S4**

*Cook's Distance Sensitivity Analysis of Negative Emotionality and BIS*

| **Psychological measure** | **Primary N** | **Primary B** | **Primary robust p** | **Sensitivity N** | **Sensitivity B** | **Sensitivity robust p** |
| --- | --- | --- | --- | --- | --- | --- |
| Negative emotionality | 144 | .378 | .011 | 135 | .391 | .003 |
| BIS | 144 | .789 | .014 | 133 | .714 | .002 |

*Note.* Negative emotionality and BIS retained similar positive coefficients and remained statistically significant after the model-specific Cook's distance flagged cases were excluded.
